# Assessment of tumor depth in oral tongue squamous cell carcinoma with multiparametric MRI: correlation with pathology

**DOI:** 10.1007/s00330-021-08148-6

**Published:** 2021-07-13

**Authors:** Weiqing Tang, Ying Wang, Ying Yuan, Xiaofeng Tao

**Affiliations:** 1 Department of Radiology, Shanghai Ninth People’s Hospital, Shanghai Jiao Tong University School of Medicine, No.639 Zhizaoju Road, Shanghai, China; 2 Department of Otorhinolaryngology Head and Neck Surgery, Shanghai Children’s Hospital, Shanghai Jiao Tong University, Shanghai, China

**Keywords:** Multiparametric MRI, Oral tongue squamous cell carcinoma, Neoplasm Invasion

## Abstract

**Objectives:**

To compare the correlation of depth of invasion (DOI) measured on multiple magnetic resonance imaging (MRI) sequences and pathological DOI, in order to determine the optimal MRI sequence for measurement.

**Methods:**

A total of 122 oral tongue squamous cell carcinoma (OTSCC) patients were retrospectively analyzed, who had received preoperative MRI and surgical resection. DOIs measured on fat-suppressed T2-weighted imaging (T2WI), diffusion-weighted imaging (DWI), dynamic enhanced-T1 high-resolution insotropic volume examination (e-THRIVE), and contrast-enhanced fat-suppressed T1WI (CE-T1WI) were respectively compared to those measured in pathologic specimens. The cutoff value of the best correlated MRI sequence was determined, and the T staging accuracy of MRI-derived DOI was evaluated.

**Results:**

DOI derived from e-THRIVE showed the best correlation (r = 0.936, *p* < 0.001) with pathological DOI. The area under the curve values of MRI-derived DOI distinguishing T1 stage from T2 stage and distinguishing T2 stage from T3 stage were 0.969 and 0.974, respectively. The T staging criteria of MRI-derived DOI were 6.2 mm and 11.4 mm, with a staging accuracy of 86.9% compared to pathological DOI criteria of 5 mm and 10 mm.

**Conclusion:**

E-THRIVE was the optimal MR sequence to measure the MR-derived DOI, and DOI derived from e-THRIVE could serve as a potential cut-off value as a clinical T staging indicator of OTSCC.

**Key Points:**

*• Multiparametric MRI helps radiologists to assess the neoplasm invasion in patients with oral tongue squamous cell carcinoma.*

*• Retrospective study indicated that measurement was most accurate on enhanced-T1 high-resolution insotropic volume examination dynamic contrast enhancement images.*

*• T staging of oral tongue squamous cell carcinoma was accurate according to the dynamic contrast enhancement MRI-derived depth of invasion.*

## Introduction

Oral cavity squamous cell carcinoma, the eighth most common cancer worldwide, mostly occurs in the anterior 2/3 of the tongue [1, 2]. Oral tongue squamous cell carcinoma (OTSCC) has a relatively unfavorable prognosis due to the presence of a well-developed lympho-vascular system and lack of a strong barrier for preventing tumor propagation [3]. Therefore, surgery of OTSCC requires assessment of the tumor margin and relatively wide local excision to reduce recurrence.

Depth of invasion (DOI), defined as the distance from the reconstructed mucosal surface or basement membrane to the deepest level of invasion, is essential for obtaining sufficiently deep cancer-free margins. Previous studies indicated that DOI was an important independent prognostic factor for lymph node metastasis and survival in patients with oral cancer [4–7]. According to the eighth edition of the American Joint Committee on Cancer (AJCC) staging manual, histopathological DOI of oral cancer greater than 5 mm and 10 mm was recommended as the golden standard threshold for T staging [8]. As a result, T staging of cancer and treatment options would be changed due to millimeter-scale errors in evaluation of DOI.

Due to its excellent soft tissue resolution, magnetic resonance imaging (MRI) is widely applied in the assessment of clinical DOI of OTSCC [9–17]. However, MRI-derived DOI was statistically larger than histopathological DOI [9, 13–15] due to peritumor edema or inflammation, which might lead to overestimated clinical T staging. Consensus has not been reached on which MRI sequence was suitable for DOI measurement. Some researchers suggested that the T2-weighted imaging (T2WI) was better for the measurements of DOI [9, 18], while others performed the measurement on contrast enhanced T1WI (CE-T1WI) [11–13]. Moreover, with the rapid development of technologies, MRI sequences such as dynamic contrast-enhanced sequences or diffusion-weighted imaging (DWI) were applied [11, 12]. However, the abilities of different MRI sequences on measurement of DOI were rarely reported. Therefore, the current study aimed to evaluate and compare the accuracy of multiple MRI sequences in estimating DOI of OTSCC, in order to determine the optimal sequence for clinical DOI evaluation. The best cutoff value, or MRI-based T staging criteria, was also determined to establish clinical applicable T staging of OTSCC.

## Methods and materials

The study was approved by the institutional review board of our institute and the informed consent was waived for using MRI images and pathological data of the patients. A retrospective analysis was performed on patients who underwent curative surgery for newly diagnosed OTSCC at our institution from January 2016 to December 2019. Inclusion criteria were (1) patients with OTSCC who underwent MRI examinations within 2 weeks before surgery and (2) the lesions were pathological confirmed as OTSCC and pathological DOI was measured. Exclusion criteria were as follows: (1) patients had other head and neck tumors previously; (2) patients who had received any treatment (including biopsy, surgery, radiotherapy, or chemotherapy) for tongue cancer; and (3) poor MRI quality due to various factors such as the movement or the presence of artificial implants.

MRI examinations were performed on a 3.0-T MRI scanner (Ingenia, Philips Medical Systems) with a 16-channel head and neck coil. The MRI sequences included axial T1WI, axial T2WI and coronal T2WI, DWI, dynamic contrast-enhanced sequence (enhanced-T1 high resolution insotropic volume examination [e-THRIVE]), and axial and coronal CE-T1WI. Spectral presaturation with inversion recovery was applied in the fat-suppressed sequences. During DWI, a single-shot echo sequence was used to collect images with *b* values of 0 s/mm^2^ and 800 s/mm^2^. After injection of the Gadopentetate dimeglumine (Gd-DPTA) (Magnevist, Bayer Schering Pharma AG), e-THRIVE and CE-T1WI were obtained. The contrast agent was injected by automatic syringe with a dosage of 0.1 mmol/kg at the injection rate of 3 mL/s. The e-THRIVE was repeated eight times (30 s for each scanning time) on the axial plane after the end of contrast medium injection, with a total scanning time of 240 s. Detail parameters of each sequence are shown in Table 1.

**Table 1:** Parameters of MRI sequences

Sequence	T1WI	T2WI	T2WI	DWI	e-THRIVE	CE-T1WI	CE-T1WI
Plane	Axial	Axial	Coronal	Axial	Axial	Axial	Coronal
TR (ms)	615	3200	3000	2255	5.5	580	590
TE (ms)	18	85	80	65	1.7	15	16
Thickness/gap (mm)	3/0.3	3/0.3	4/0.4	3/0.3	3/0	3/0.3	4/0.4
Slices	32	32	24	18	18	32	24
Matrix	300 × 200	300 × 200	368 × 210	88 × 100	300 × 200	300 × 200	300 × 200
FOV (cm)	24 × 20	24 × 20	22 × 20	24 × 20	24 × 20	24 × 20	22 × 20
NSA	1.5	2	2	5	1	1.5	1.5
Fat suppression	No	Both	No	Yes	Yes	Both	Yes

*Abbreviations*: *TR* repetition time, *TE* echo time, *T1WI* T1-weighted imaging, *T2WI* T2-weighted imaging, *DWI* diffusion-weighted imaging, *e-THRIVE* enhanced-T1 high-resolution insotropic volume examination, *CE* contrast-enhanced, *NSA* number of signal averages

**Fig. 1:**
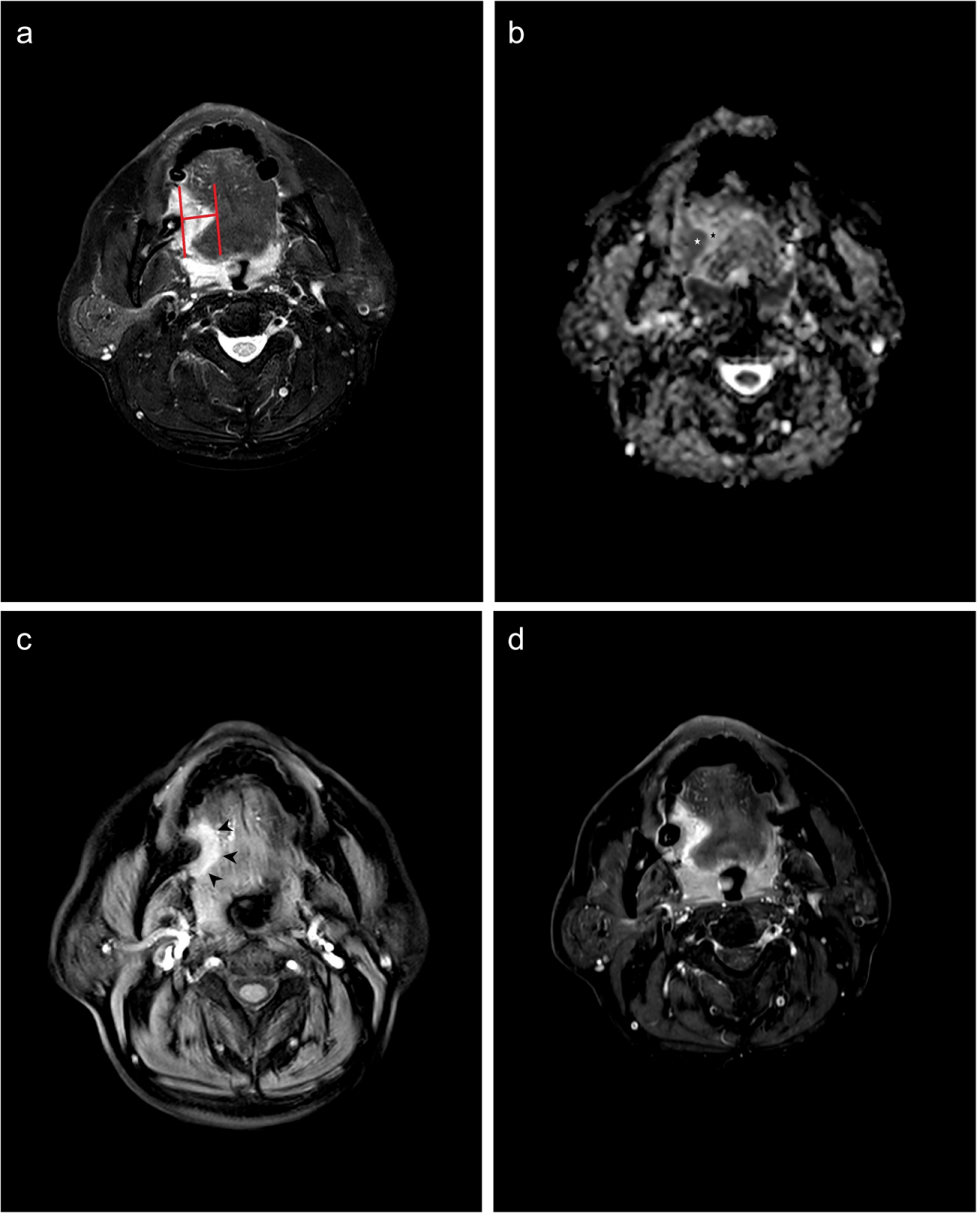
A 55-year-old male with OTSCC in the right border of tongue. The pathological DOI was 7.0 mm. The DOIs measured on T2WI, DWI, e-THRIVE, and CE T1WI were 15.0 mm, 8.1 mm, 8.6 mm, and 15.6 mm, respectively. **a** The schematic diagram of DOI measurement on T2WI. **b** On ADC map, the tumor showed hypointensity (white star) and edema showed hyperintensity (black star). **c** The tumor showed significant enhancement on the e-THRIVE. The margin of tumor was well-defined (black arrow). **d** Both tumor and perilesional edema showed hyperintensity on contrast-enhanced T1WI

All MRI-derived DOIs were independently measured by two experienced head and neck radiologists (W.T. with 7 years of experience and Y.Y. with 10 years of experience). The largest section of tumor on axial images was selected for measurement. Two parallel lines were drawn on the mucosa surface and the deepest point of the tumor, respectively, and then the distance between the two parallel lines was measured as the sequence specific DOI (Fig. 1). For exogenous tumors, the part above the mucosal surface was neglected, and for ulcerative tumors, the invaginated part was added [9]. The measurement of each sequence was performed on fat-suppressed T2WI, DWI, e-THRIVE, and fat-suppressed CE-T1WI, respectively (Fig. 1). All radiologists were blinded to clinical and pathological data. DOIs derived from DWI and e-THRIVE were measured on the apparent dispersion coefficient (ADC) maps and the early post-contrast images (60 s after injection), respectively.

The intraclass correlation coefficient (ICC) based on a two-way random effect model was applied to evaluate the inter-rater reliability of MRI based measurements. The MRI-derived DOIs on each sequence were compared with pathological DOI by paired *t*-test, and the correlations of MRI-derived DOI with pathological DOI were evaluated by Pearson’s correlation coefficient. The sequence with the best correlation was selected for further receiver operation characteristic (ROC) curve analysis. The staging accuracy was assessed by ROC analysis with the area under the curve (AUC) value, and the optimal cutoff values for MRI-based clinical T staging were determined. SPSS version 21.0 was used for all statistical analyses, and a two-paired *p* < 0.05 was considered to be of statistical significance.

## Results

A total of 122 patients fulfilled the inclusion and exclusion criteria, including 64% (78/122) of males and 46% (44/122) of females. The ages of included cases ranged from 28 to 76 years (median age: 62 years). The surgical margin was proved to be negative in all cases. Based on the eighth edition of the AJCC staging, 30.3% (37/122), 36.1% (44/122), and 33.6% (41/122) of cases were respectively classified as pathological T1 stage, T2 stage, and T3 stage. The border of the tongue was the most common tumor site, accounting for 77.9% (95/122) of the tumors, followed by ventral and dorsal surfaces, accounting for 19.6% (25/122) and 2.5% (3/122) of the tumors, respectively. For morphological type, most tumors (83.6%, 102/122) were presented as endophytic mass on MR images, with the remaining tumors presenting as exogenous type (9.8%, 12/122) and ulcerative type (6.6%, 8/122).

The tumor and the peritumoral inflammation can be distinguished on DWI and e-THRIVE (Fig. 1). The inter-rater reproducibility of DOI measured on T2WI (ICC = 0.902, *p* < 0.001), DWI (ICC = 0.899, *p* < 0.001), e-THRIVE (ICC = 0.913, *p* < 0.001), and CE-T1WI (ICC = 0.921, *p* < 0.001) were all excellent. The DOIs measured on T2WI, DWI, e-THRIVE, and CE-T1WI were 12.67 ± 6.71 mm (mean ± standard deviation [SD]), 10.98 ± 6.24 mm, 11.12 ± 6.76 mm and 12.55 ± 6.73 mm, respectively, which were all statistically larger than pathological DOIs (9.33 ± 5.61 mm) (*p* < 0.001). DOIs measured in T2WI, DWI, e-THRIVE, and CE-T1WI were smaller than pathological DOIs in 6.6% (8/122), 13.1% (16/122), 5.7% (7/122), and 4.0% (6/122) of the cases, respectively. Measurement on DWI was unsuccessful in five T1 stage tumors due to artifacts.

**Fig. 2:**
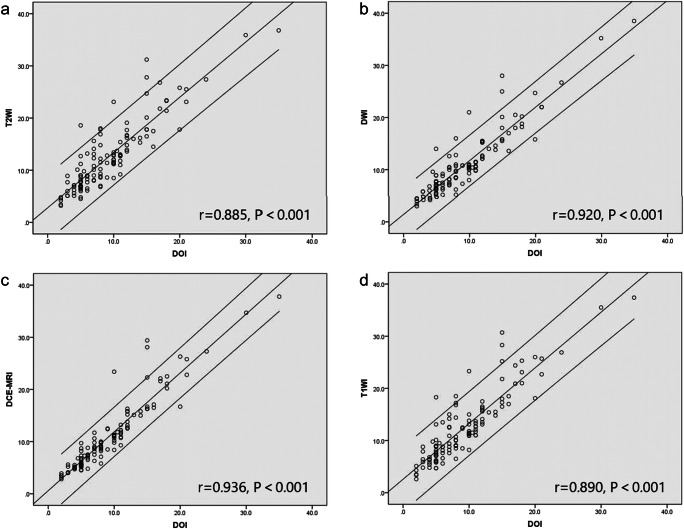
Correlation between each MRI sequence based DOI and pathological DOI. **a** T2WI. **b** DWI. **c** E-THRIVE. **d** CE T1WI

**Fig. 3:**
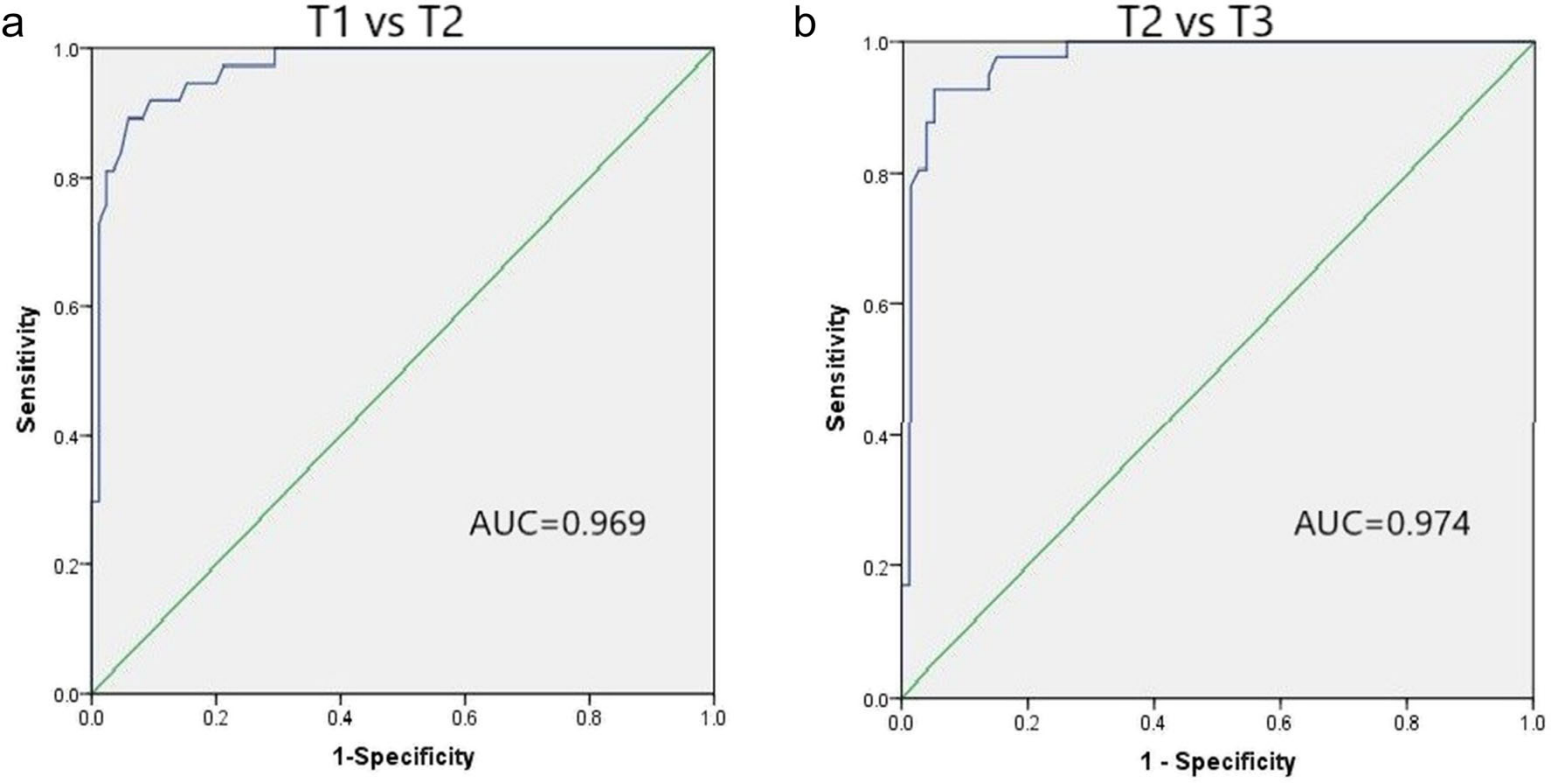
ROCs for the differentiating performance of e-THRIVE-derived DOI among the defined groups of OTSCC based on the eighth edition of the AJCC staging. **a** T1 stage vs. T2 stage. **b** T2 stage vs. T3 stage

Pearson’s coefficient of MRI-derived DOI and pathological DOI is shown in Table 2 and Fig. 2. E-THRIVE-derived DOI showed the highest Pearson’s coefficient (*r* = 0.936, *p* < 0.001), followed by DWI-derived DOI (*r* = 0.920, *p* < 0.001). The subgroup analysis stratified by pathological T staging and morphology of tumor also indicated that Pearson’s coefficients of e-THRIVE-derived DOI and pathological DOI were the highest, which ranged from 0.801 to 0.947 (Table 2). The subgroup correlation results are listed in Table 2. The area under the ROC curve (AUC) of distinguishing T1/T2 stage and distinguishing T2/T3 stage was 0.969 and 0.974, respectively (Fig. 3). According to the ROC curve, the e-THRIVE-derived DOI value of 6.2 mm and 11.4 mm can serve as the cutoff value for T staging of OTSCC (T1: DOI ≤ 6.2, T2: 6.2 mm < DOI ≤ 11.4 mm, T3: DOI > 11.4 mm). This e-THRIVE-derived T staging system successfully classified 86.9% (106/122) of the patients when compared to pathological DOI, with 9.0% (11/122) of cases overestimated and 4.1% (5/122) underestimated (Kendall’s tau-b = 0.87, *p* < 0.001) (Table 3).

**Table 2:** The Pearson’s coefficient of MRI-derived DOI and pathological DOI

	Pearson’s coefficient (*p*)			
	T2WI	DWI	e-THRIVE	CE T1WI
Overall	0.885 (< 0.001)	0.920 (< 0.001)	0.936 (< 0.001)	0.890 (< 0.001)
Pathological T stage				
Early stage (T1–T2)	0.641 (< 0.001)	0.700 (< 0.001)	0.801 (< 0.001)	0.649 (< 0.001)
Advanced stage (T3)	0.838 (< 0.001)	0.895 (< 0.001)	0.877 (< 0.001)	0.857 (< 0.001)
Morphology				
Endophytic	0.887 (< 0.001)	0.926 (< 0.001)	0.942 (< 0.001)	0.888 (< 0.001)
Exophytic	0.911 (< 0.001)	0.936 (< 0.001)	0.947 (< 0.001)	0.940 (< 0.001)
Ulcerated	0.825 (= 0.008)	0.830 (= 0.006)	0.846 (= 0.006)	0.806 (= 0.005)

*Abbreviations*: *T1WI* T1-weighted imaging, *T2WI* T2-weighted imaging, *DWI* diffusion-weighted imaging, *e-THRIVE* enhanced-T1 high-resolution insotropic volume examination, *CE* contrast-enhanced

**Table 3:** Accuracy of clinical T staging based on e-THRIVE-derived DOI

e-THRIVE-derived DOI	Pathological DOI			Total
	≤ 5 mm (T1)	5 mm < DOI ≤ 10 mm (T2)	> 10 mm (T3)	
≤ 6.2 mm (T1)	30	2	0	32
6.2 mm < DOI ≤ 11.4 mm (T2)	7	38	3	48
> 11.4 mm (T3)	0	4	38	42
Total	37	44	41	122

*Abbreviations*: *e-THRIVE* enhanced-T1 high-resolution insotropic volume examination, *DOI* depth of invasion

## Discussion

The eighth edition of the AJCC staging manual added histopathological DOI cutoff values of ≤ 5 mm, > 5 mm but ≤ 10 mm, and > 10 mm as a new staging criterion to the T stage of oral cancer [8]. Therefore, T staging of OTSCC would be changed by even millimeter errors of DOI. Although accurate radiological measurements of DOI can help a surgeon choose the proper therapeutic planning, there was no radiological criteria established for determining it.

In this study, four kinds of MR sequences were applied in the measurement of DOI. We found significant correlation between MRI-derived DOI on each sequence and pathological DOI, the partial results of which are consistent with previous studies [11, 13, 17]. Although correlations are interesting, a factor warranting greater attention is that MRI-derived DOI was significantly larger than histopathological DOI, which led to the overestimating of T stages [12, 13]. In addition, the exact value of the difference was not in consensus, which was up to 3.5 mm [9]. A similar difference was also found in the present study. Therefore, we should first consider the difference between MRI-derived DOI and histopathological DOI in order to make the prediction of T staging more accurate.

Two possible explanations were proposed for the larger size of MRI-derived DOI. One is the shrinkage of soft tissue during formalin fixation. It has been reported that the shrinkage ranged from 14.9 to 23.9% of oral cavity tissues [19]. The scale in microscopes is usually adjusted according to the supposed shrinkage effect. The other factor is peritumoral edema and reactive inflammation providing the overestimation of the MRI-derived DOI. However, some forms of chronic inflammatory, reactive conditions of the oral cavity are nearly indistinguishable from the malignant tumors, both clinically and radiologically [20]. The tumor and the peritumoral inflammation were shown to have similar signal intensity on T1WI or T2WI [21]. However, there was disagreement on avoiding this factor, which mainly focused on the choice of MR sequence. Some researchers thought that T2WI was better for measurement than T1WI [9, 18]; others had the opposite view [13]. Baba et al [16] reported that the measurement of CE-T1WI-derived DOI was slightly more accurate than that of T2WI-derived DOI for a better correlation between CE-T1WI-derived DOI and histopathological DOI. Another point of view was that optimal MR sequence differed for each patient [14, 15]. In this study, we found that measurement of DOI on e-THRIVE was the most accurate, followed by that on DWI. Accuracy of measurement on T2WI and CE-T1WI were similar, but both were lower than that on e-THRIVE and DWI.

It has a nearly linear relationship between contrast concentration and signal intensity on dynamic contrast-enhanced sequence, which are commonly used in the diagnosis of head and neck lesions [22–24]. Compared to CE-T1WI, measurements on e-THRIVE are not significantly influenced by the partial volume effect for a fast T1 gradient-echo sequence routinely used in a three-dimensional mode. In addition, due to the fact that the signal of a malignant tumor rises faster than that of a benign lesion and 59.58 s was the average time when signal intensity of malignant tumor reached the peak [24], it is possible to avoid the error of DOI related to the peritumoral edema and reactive inflammation by performing measurements at early post-contrast images (< 60 s) [12, 24]. We did not measure the DOI on other phase dynamic sequence except for the early phase, since previous study indicated that morphology of lesions was similar on the later phase dynamic sequences [25], in which peritumoral edema or inflammation might not be distinguished from tumor [12]. To our knowledge, Vidiri et al first applied a similar sequence in the measurement of DOI [12]. Their study showed high agreement between MRI-derived DOI and pathological DOI. However, their study sample was small, and the accuracy of measurements on early T stage OTSCC was not fully discussed. Furthermore, other MR sequences were not included for comparison of the accuracy. Our study indicated that e-THRIVE-derived DOI had the highest correlation with pathological DOI. According to the ROC curve, 6.2 mm and 11.4 mm were the probable cutoff values for the MR staging criteria. With these cutoff values as the diagnostic criteria, the accuracy of MRI in T stage of OTSCC was 86.9% compared with pathology. Although the correlation between e-THRIVE-derived DOI and pathological DOI was reduced to 0.801 in patients with an early T stage (T1 or T2) lesion, it was still highest compared with other sequences. It should be noted that measurements at early post-contrast images might underestimate pathological DOI in some lesions with diffused margin [25]. Our study showed that only 5.7% (7/122) of e-THRIVE-derived DOI was smaller than pathological DOI, which indicated that measurement on e-THRIVE was accurate.

DWI is another functional MR sequence that is commonly used in the diagnosis of head and neck tumors. DWI has great ability to distinguish between tumor and peritumoral inflammation [26] and is helpful in staging bladder tumors [27]. To our knowledge, this is the first study that applied DWI at the stage of OTSCC. Results of our study showed that DWI-derived DOI was also correlated with pathological DOI. Compared with e-THRIVE, the advantage of DWI is that it can be performed without administering a contrast agent, which means lower cost, faster scanning time, and a decreased risk of complications. One concern is that even with the use of improved echo-planar imaging technology, dedicated coils, and dedicated sequence optimization, a 4.1% (5/122) failure rate of measurement on DWI still existed in the current study. Two possible reasons for this were assumed after a review of all the failed cases. One is the size of the lesions, because pathological DOI of all failed cases was smaller than 5 mm. The other is metal- or motion-related artifact generation, which affected imaging quality. Barring these reasons, our study still indicated that DWI is the first choice for measurement in the cases without administration of a contrast agent.

To measure the DOI accurately, it is important to develop the optimal parameters of the MR sequence. Mao et al [9] used 1-mm thin-slice scanning in their measurements, which improved the space resolution ratio of MRI. However, thinner-slice MR scanning was useless in the improvement of soft tissue resolution. In addition, more scanning time was needed to achieve 1-mm thin-slice scanning, which may lead to more motion-related artifacts. Therefore, to balance the image quality and spatial resolution, 3-mm slice scanning was performed in this study.

There were some limitations to this study. Potential bias in the interpretations of images cannot be entirely excluded because two readers reviewed all MR sequences. With that in mind, precautions were taken to minimize the effects of double reading. Moreover, the study results suggest that measurement on e-THRIVE or DWI was not biased by other sequences for signal intensity of the same lesions performing differently on each sequence (Fig. 1). The correlation between the DOI and the status of the lymph nodes at diagnosis and between the DOI and loco-regional recurrence at the follow-up was not evaluated in this study. These correlations and further studies are currently in progress.

In conclusion, this study indicated the highest correlation between the e-THRIVE-derived DOI and the pathological DOI. For MRI-derived DOI, measurement on the early e-THRIVE was the optimized method in the T staging of OTSCC. The ROC curve in this study revealed that MRI-measured depths of invasion of 6.2 mm and 11.4 mm were used as staging criteria of OTSCC, with a diagnostic accuracy of 86.9%.

## Abbreviations

ADC: Apparent dispersion coefficient
AJCC: American Joint Committee on Cancer
AUC: Area under the ROC curve
CE: Contrast enhanced
DOI: Depth of invasion
DWI: Diffusion-weighted images
e-THRIVE: Enhanced-T1 high-resolution insotropic volume examination
ICC: Intraclass correlation coefficient
MRI: Magnetic resonance imaging
OTSCC: Oral tongue squamous cell carcinoma
ROC: Receiver operation characteristic
T2WI: T2-weighted imaging

## Declarations

### Guarantor

The scientific guarantor of this publication is Ying Yuan.

### Statistics and biometry

No complex statistical methods were necessary for this paper.

### Methodology

• retrospective

• diagnostic or prognostic study

• performed at one institution

## References

[1] Ettinger KS, Ganry L, Fernandes RP (2019). Oral cavity cancer. Oral Maxillofac Surg Clin North Am.

[2] Siegel RL, Miller KD, Jemal A (2018). Cancer statistics, 2018. CA Cancer J Clin.

[3] Koo BS, Lim YC, Lee JS, Choi EC (2006). Recurrence and salvage treatment of squamous cell carcinoma of the oral cavity. Oral Oncol.

[4] Ling W, Mijiti A, Moming A (2013). Survival pattern and prognostic factors of patients with squamous cell carcinoma of the tongue: a retrospective analysis of 210 cases. J Oral Maxillofac Surg.

[5] Tan WJ, Chia CS, Tan HK, Soo KC, Iyer NG (2012). Prognostic significance of invasion depth in oral tongue squamous cell carcinoma. ORL J Otorhinolaryngol Relat Spec.

[6] Mücke T, Kanatas A, Ritschl LM (2016). Tumor thickness and risk of lymph node metastasis in patients with squamous cell carcinoma of the tongue. Oral Oncol.

[7] Ebrahimi A, Gil Z, Amit M (2014). Primary tumor staging for oral cancer and a proposed modification incorporating depth of invasion: an international multicenter retrospective study. JAMA Otolaryngol Head Neck Surg.

[8] Lydiatt WM, Patel SG, O ' Sullivan B et al (2017) Head and neck cancers-major changes in the American Joint Committee on cancer eighth edition cancer staging manual. CA Cancer J Clin 67:122–137 10.3322/caac.21389 28128848

[9] Mao MH, Wang S, Feng ZE (2019). Accuracy of magnetic resonance imaging in evaluating the depth of invasion of tongue cancer. A prospective cohort study. Oral Oncol.

[10] Alsaffar HA, Goldstein DP, King EV (2016). Correlation between clinical and MRI assessment of depth of invasion in oral tongue squamous cell carcinoma. J Otolaryngol Head Neck Surg.

[11] Huopainen P, Jouhi L, Hagstrom J, Apajalahti S (2020). MRI correlates to histopathological data in oral tongue squamous cell carcinoma diagnostics. Acta Odontol Scand.

[12] Vidiri A, Panfili M, Boellis A (2019). The role of MRI-derived depth of invasion in staging oral tongue squamous cell carcinoma: inter-reader and radiological–pathological agreement. Acta Radiol.

[13] Fu JY, Zhu L, Li J et al (2020) Assessing the magnetic resonance imaging in determine the depth of invasion of tongue cancer. Oral Dis. 10.1111/odi.13579 10.1111/odi.13579 32731298

[14] Murakami R, Shiraishi S, Yoshida R (2019). Reliability of MRI-derived depth of invasion of oral tongue cancer. Acad Radiol.

[15] Noorlag R, Klein Nulent TJW, Delwel VEJ (2020). Assessment of tumour depth in early tongue cancer: accuracy of MRI and intraoral ultrasound. Oral Oncol.

[16] Baba A, Okuyama Y, Yamauchi H (2019). Magnetic resonance imaging findings of styloglossus and hyoglossus muscle invasion: relationship to depth of invasion and clinical significance as a predictor of advisability of elective neck dissection in node negative oral tongue cancer. Eur J Radiol.

[17] Park JO, Jung SL, Joo YH, Jung CK, Cho KJ, Kim MS (2011). Diagnostic accuracy of magnetic resonance imaging (MRI) in the assessment of tumor invasion depth in oral/oropharyngeal cancer. Oral Oncol.

[18] Kwon M, Moon H, Nam SY (2016). Clinical significance of three-dimensional measurement of tumour thickness on magnetic resonance imaging in patients with oral tongue squamous cell carcinoma. Eur Radiol.

[19] Pangare TB, Waknis PP, Bawane SS, Patil MN, Wadhera S, Patowary PB (2017). Effect of formalin fixation on surgical margins in patients with oral squamous cell carcinoma. J Oral Maxillofac Surg.

[20] Kim ST, Kim HJ, Park IS, Park SW, Kim WH, Kim YM (2005). Chronic, reactive conditions of the oral cavity simulating mucosal carcinomas. Clin Imaging.

[21] Imaizumi A, Yoshino N, Yamada I (2006). A potential pitfall of MR imaging for assessing mandibular invasion of squamous cell carcinoma in the oral cavity. AJNR Am J Neuroradiol.

[22] Marzi S, Piludu F, Forina C (2017). Correlation study between intravoxel incoherent motion MRI and dynamic contrast-enhanced MRI in head and neck squamous cell carcinoma: evaluation in primary tumors and metastatic nodes. Magn Reson Imaging.

[23] Yan S, Wang Z, Li L (2016). Characterization of cervical lymph nodes using DCE-MRI: differentiation between metastases from SCC of head and neck and benign lymph nodes. Clin Hemorheol Microcirc.

[24] Choi YJ, Lee JH, Sung YS (2016). Value of dynamic contrast-enhanced MRI to detect local tumor recurrence in primary head and neck cancer patients. Medicine (Baltimore).

[25] Ariyoshi Y, Shimahara M (2006). Relationships between dynamic contrast enhanced MRI findings and pattern of invasion for tongue carcinoma. Oncol Rep.

[26] Thoeny HC, De Keyzer F, King AD (2012). Diffusion-weighted MR imaging in the head and neck. Radiology.

[27] Takeuchi M, Sasaki S, Ito M (2009). Urinary bladder cancer: diffusion-weighted MR imaging—accuracy for diagnosing T stage and estimating histologic grade. Radiology.

